# Plasmacytoma of tonsil diagnosed by fine-needle aspiration cytology

**DOI:** 10.4103/0970-9371.71875

**Published:** 2010-07

**Authors:** Ramachandra V Bhat, KM Prathima, Harendra ML Kumar, GK Narayana

**Affiliations:** Department of Pathology, Aarupadai Veedu Medical College, Kirumampakkam, Pondicherry, India; 1 Deparment of Pathology, Sri Devaraj Urs Medical College, Kolar, Karnataka, India; 2 Department of ENT, Sri Devaraj Urs Medical College, Kolar, Karnataka, India

**Keywords:** Plasmacytoma, tonsil, FNAC

## Abstract

Extramedullary plasmacytoma of tonsil is rare. Even though biopsy is necessary for final diagnosis, fine-needle aspiration cytology (FNAC) can provide useful information in the management of such cases. We report a case of plasmacytoma of tonsil diagnosed by FNAC in a 43-year-old man who presented with a swelling in the right tonsillar area. FNAC smears revealed sheets of plasma cells at various stages of maturation. Subsequent histopathological and immunohistochemical studies confirmed the diagnosis of plasmacytoma. This case is reported for the rarity of site for extramedullary plasmacytoma and to highlight the usefulness of FNAC in lesions of tonsil.

## Introduction

Plasmacytoma is a localized plasma cell neoplasm known to occur both inside and outside the bone. Extramedullary plasmacytomas are rare and are found at various sites, such as nasal fossa, oral cavity, lymph node, breast and soft tissue.[1]

Extramedullary plasmacytoma of tonsil is rare which cause tonsillar asymmetry and can be clinically mistaken for malignancy. Fine-needle aspiration cytology (FNAC) is a minimally invasive procedure and is helpful in preoperative diagnosis of such lesions.

## Case Report

A 43-year-old man came to the ENT department of our hospital with a history of discomfort during swallowing of 3-months duration. He also noticed a swelling in the right tonsillar area. On clinical examination there was a firm swelling in the right tonsillar area measuring 4×3 cm, surface was smooth and congested. Oral cavity and left tonsillar area were normal. A clinical diagnosis of tonsillar cyst or carcinoma was made. FNAC of the lesion was performed and smears were stained with Papanicolaou stain (PAP stain) and May–Grünwald–Giemsa (MGG) stain.

**Figure 1:**
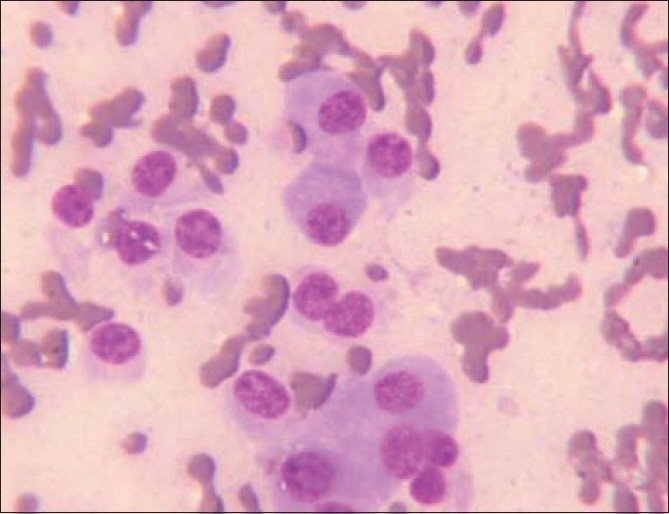
Cytology smears showing plasma cells at different stages of maturation (MGG, ×400)

The aspiration smears were cellular and revealed plasma cells having eccentric nuclei with abundant cytoplasm. Few bi- and tri-nucleated forms and also mononuclear immature cells with prominent nucleoli were seen in a hemorrhagic background [Figure 1].

Cytological diagnosis of extramedullary plasmacytoma was offered and investigations such as blood and urine examination, radiological survey, bone marrow aspiration study and serum electrophoresis were suggested to rule out multiple myeloma.

Other investigations subsequently done, such as hemoglobin, total and differential count and peripheral smear examination, were within normal range. Erythrocyte sedimentation rate was 40 mm at the end of 1 hour, urine routine examination was within normal limits and Bence–Jones protein was negative. Radiographs of the pelvis, skull and ribs were normal. Bone marrow aspiration study was within normal limits and it showed only 4% plasma cells.

**Figure 2:**
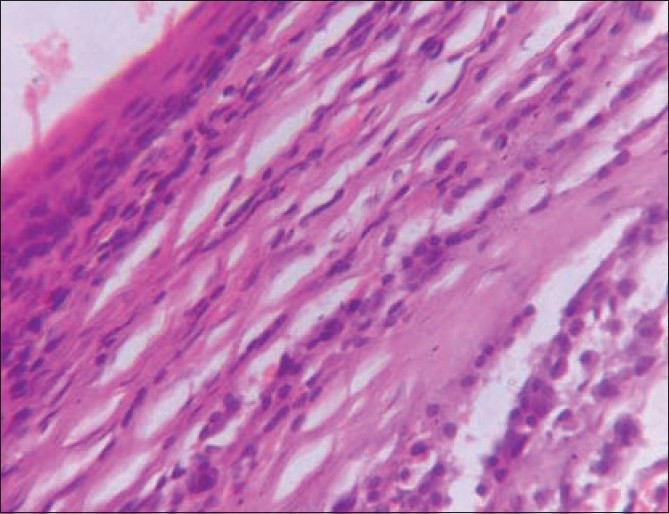
Tissue section from tonsil showing infiltration by neoplastic plasma cells (H and E, ×100)

Right-sided tonsillectomy was done and specimen was sent for histopathological examination. Grossly the specimen was a single grey-brown oval mass measuring 2.5×1.5×1.5 cm. Cut section showed solid grey-white area with foci of hemorrhage. Microscopically, the sections showed tonsillar tissue with sub-epithelial lymphoid aggregates with a well-circumscribed tumor composed of sheets of plasma cells [Figure 2] with a few bi- and tri-nucleated cells. The tumor cells were separated by thin fibrous septa. Small foci of eosinophilic substance that was negative for amyloid by Congo red stain were also seen. Immunohistochemical study showed kappa light chain restriction in the neoplastic cells, which confirmed the diagnosis of plasmacytoma.

## Discussion

FNAC has become an important preoperative diagnostic procedure for any palpable swelling, but FNAC of tonsillar mass is infrequently done. With the assistance of ENT surgeon, FNAC of tonsil can be done, and it is a safe, cost-effective and minimally invasive procedure.[2]

Extramedullary plasmacytoma of tonsil is rare. Clinically it may be mistaken for squamous cell carcinoma or lymphoma.[3] In this case it was clinically diagnosed as tonsil cyst or malignancy.

FNAC yielded good cellularity and the cytological findings were very characteristic, showing numerous plasma cells at various stages of maturation similar to the observations of other authors.[1,4] Histological and immunohistochemical studies confirmed the cytological diagnosis. A similar case was reported by Sakai *et al*.,[5] in a 53-year-old man, in whom they noticed bi-nucleated cells and atypical cells in cytology smears.

Tonsillar plasmacytoma may show crystal inclusions in the cytoplasm of plasma cells[6] or may be associated with AL (Amyloid Light chain) amyloidosis, ossification and multinucleated giant cells.[7] No such findings were noted in our case. The overall prognosis in extra-medullary plasmacytoma is excellent;[4] our patient is asymptomatic 2 years after diagnosis.

In conclusion, this is a rare case of extramedullary plasmacytoma of tonsil. FNAC of tonsil can be done safely and is useful for preoperative diagnosis of tonsillar tumors. In case of extramedullary plasmacytoma, one has to rule out the possibility of multiple myeloma by carrying out other relevant investigations.
